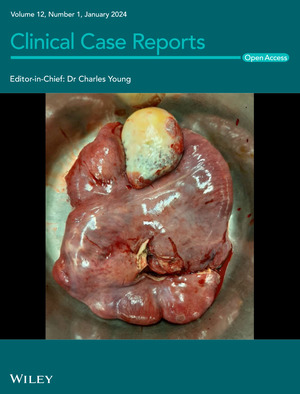# Cover Image

**DOI:** 10.1002/ccr3.8466

**Published:** 2024-01-21

**Authors:** Ali Tajaddini, Mohammadmehdi Fallahi, Hoda Haghshenas, Soheila‐sadat Nourmohammadi, Leila Ghahramani, Reza Shahriarirad

## Abstract

The cover image is based on the Case Report *Primary abdominal cocoon syndrome manifesting with Chilaiditi syndrome and intestinal obstruction: A case report* by Ali Tajaddini et al., https://doi.org/10.1002/ccr3.8363